# Prevalence of ApoE Alleles in a Spanish Population of Patients with a Clinical Diagnosis of Alzheimer’s Disease: An Observational Case-Control Study

**DOI:** 10.3390/medicina60121941

**Published:** 2024-11-25

**Authors:** Laura Bello-Corral, Jesús Seco-Calvo, Angela Molina Fresno, Ana Isabel González, Ana Llorente, Diego Fernández-Lázaro, Leticia Sánchez-Valdeón

**Affiliations:** 1 Health Research Nursing Group (GREIS), University of Leon, 24071 Leon, Spain; lbelc@unileon.es (L.B.-C.); lsanv@unileon.es (L.S.-V.); 2 Department of Nursing and Physiotherapy, University of Leon, 24071 Leon, Spain; 3 Institute of Biomedicine, University of León, 24071 Leon, Spain; 4 Department of Physiology, University of the Basque Country, 48940 Leioa, Spain; 5 Faculty of Chemistry, Rovira i Virgili University, 43007 Tarragona, Spain; angela.molina@estudiants.urv.cat; 6 Genetics Area, Department of Molecular Biology, University of León, 24071 Leon, Spain; ana-isabel.gonzalez@unileon.es (A.I.G.); ana.llorente@unileon.es (A.L.); 7 Department of Cellular Biology, Genetics, Histology and Pharmacology, Faculty of Health Sciences, University of Valladolid, Campus of Soria, 42004 Soria, Spain; 8 Neurobiology Research Group, Faculty of Medicine, University of Valladolid, 47002 Valladolid, Spain

**Keywords:** Alzheimer’s disease, ApoE polymorphism, genetic, case control, geographical distribution

## Abstract

*Background and Objectives*: Alzheimer’s dementia is a progressive neurodegenerative disease that affects memory abilities due to genetic and environmental factors. A well-known gene that influences the risk of Alzheimer’s disease is the apolipoprotein E (APOE) gene. The APOE gene is involved in the production of a protein that helps transport cholesterol and other types of fat in the bloodstream. Problems in this process are thought to contribute to the development of Alzheimer’s disease. APOE comes in several forms, which are called alleles (ε2, ε3, ε4). *Materials and Methods*: Therefore, our study aims to identify those subjects with a higher genetic risk through the polymorphism of the APOE gene, using a population screening in patients with a clinical diagnosis of AD in a region of Spain, Castilla y León, as potential biomarkers and to identify individuals at increased genetic risk by polymorphism of the APOE gene. An observational case-control study was conducted in Castilla y León (Spain). Saliva samples were collected and the ApoE gene was analyzed by PCR and agarose gel electrophoresis, respecting ethical criteria. *Results*: In the Alzheimer’s population in Castilla y León, a high prevalence of ApoE3 (74%) was found, followed by ApoE4 (22%); in addition, a higher presence of the ε4 allele was found in the Alzheimer’s disease (AD) group than in the control group. It was also observed that the ε2/ε2 genotype was not found in any individual with AD but was found in healthy subjects and that the opposite was observed for the ε4/ε4 genotype. The odds ratio (OR) indicated a risk four times greater of having AD if having the ε4 allele. *Conclusions*: The demonstrated relation between the different isoforms and the likelihood of developing AD has led to its consideration as a biomarker and a potential pre-symptomatic therapy. The molecular mechanisms that confer a disruptive and protective role to ApoE4 and ApoE2, respectively, are still being studied.

## 1. Introduction

Alzheimer’s disease (AD) is a progressive neurodegenerative pathology characterized by a series of alterations in specific regions of the brain, such as the neocortex, entorhinal area, and hippocampus [1]. Since its description in 1906 by Alois Alzheimer [2], AD has shown high prevalence and incidence rates, which may be underestimated [3]. The current and future challenge in the field of AD is the need to diagnose it in its preclinical stages, which requires the identification of disease markers during the asymptomatic phase. Early diagnosis is essential to modify the course of the disease through preventive treatments [4].

AD is considered a multifactorial pathology [5] and the underlying molecular events are still being investigated. Deposition of beta-amyloid peptide (Aβ) forming senile plaques has been observed in AD patients, as well as increased phosphorylation of the tau protein and the formation of neurofibrillary tangles [6,7]. Other factors considered as possible causes of neurodegeneration could be neuroinflammation, oxidative stress and injury to cholinergic neurons [5,8]. However, genetic factors also seem to play a major role in AD [5]. Typically, AD has been divided into early-onset AD (EOAD), related to rare autosomal forms of the disease [9], and late-onset AD (LOAD), more common and related to multiple risk genes identified by genome-wide association studies (GWASs) [10,11]. The E4 allele of the apolipoprotein E-encoding gene (APOE) has been identified as a risk factor in both EOAD and LOAD.

The APOE gene is located on the long arm of chromosome 19 and contains four exons and three introns [12]. It is a polymorphic gene with three alleles that differ by two specific amino acid isoforms that impact the function and structure of the protein [7]: ε2, ε3 and ε4. Its inheritance is codominant, resulting in six genotypes: three homozygous genotypes (ε2/ε2, ε3/ε3 and ε4/ε4) and three heterozygous (ε2/ε3, ε2/ε4 and ε3/ε4) [13]. The three resulting ApoE isoforms would be ApoE2 (cys112, cys158), ApoE3 (cys112, arg158), and ApoE4 (arg112, arg158) [14]. The ApoE protein plays an important role in lipid transport [7] and metabolism and tissue repair [9]. The difference in sequence substantially affects the functionality of the protein, resulting in a different affinity for their respective receptors [15].

Inheritance of one or two ε4 alleles has been shown to increase the risk of developing the disease by approximately three times for each copy of the ε4 allele, as well as reducing the age of onset. However, inheritance of ε2 lowers the risk and delays the age of onset [16].

How ApoE4 contributes to the development of AD is not yet fully understood, but several mechanisms have been proposed. The effect of ApoE4 on Aβ peptide accumulation is thought to be the main mechanism linking ApoE4 to the AD risk [17,18]. Fleisher et al. [18] measured the brain Aβ peptide concentrations by PET and established that those carrying the ApoE4 variant are more likely to have abnormally high levels of Aβ peptide42 (Aβ42). They also indicated that amyloid positivity on imaging appears to start at around 56 years in cognitively healthy carriers, compared to 76 years in ApoE4 non-carriers. However, Morris et al. [17] indicated that, in cognitively healthy older people, ApoE2 carriers showed a reduced peptide load. However, this may not be the only mechanism involved. Conejero et al. [19] observed that, in ApoE4 individuals, pathogenic processes such as the accumulation of Aβ peptide or hyperphosphorylated Tau protein are triggered early due to various abnormalities in signaling pathways and biological processes related to calcium control, mitochondrial function, irregularities in cell cycle regulation and apoptosis. Similarly, Reiman et al. [20] observed decreased brain activity in cognitively normal individuals carrying the ε4 allele.

Therefore, our study aims to identify those subjects with a higher genetic risk through APOE gene polymorphism, through population-based screening in patients with a clinical diagnosis of AD in a region of Spain, Castilla y León, to personalize future interventions in daycare centers for these AD patients. Also, we set out to develop a noninvasive, saliva-based biomarker for early diagnosis. Saliva will allow us to monitor patients entering our AD care centers easily. We hypothesize that the ε2 and ε4 alleles have a protective and disruptive role, respectively, so the presence of certain APOE gene polymorphisms could be a potential biomarker for the early diagnosis of AD. Thus, identifying those at higher genetic risk through the ApoE gene polymorphism could allow more effective early interventions and prevention strategies to be implemented, thereby improving how the early stages of AD are addressed.

## 2. Materials and Methods

### 2.1. Study Design

An analytical observational case-control study was carried out in the “Mensajeros de La Paz” and Asociaciones de Familiares de Enfermos de Alzheimer (AFA) residences in Castilla y León (Spain), using the Strengthening the Reporting of Observational Studies in Epidemiology (STROBE) statement as a reference (see Appendix A). This subject environment origin was chosen because of the population of users of the “Mensajeros de la Paz” and AFA homes in Castilla y León (Spain), which, due to its geographical location at a crossroads, has received many genetic contributions from northern Europe, the Mediterranean area and northern Africa.

This research adhered to the stringent ethical norms established by the Declaration of Helsinki and its subsequent amendments. This study was initiated only when permission was obtained from the Ethics Committee of the University of León (code “ETICA-ULE-021-2022”) and registered in ClinicalTrials.gov with code NCT06275243.

### 2.2. Study Cohort

The study included 511 individuals between 60 and 90 years of age, both men and women. Of these, 260 had a clinical diagnosis of AD, without visualization of plaques and tangles within the brain, by post-mortem examination or by neuroimaging, so that a definitive diagnosis was not possible, who were “*probable AD patients*” (pAD) and formed the case group, while the remaining 251 were healthy individuals and formed the control group.

All the participants underwent an initial medical examination that included a review of their medical history and approval by their physician. Throughout the study, participants were under strict health monitoring. The participants were included consecutively, from October 2022 to May 2024, immediately from the moment of clinical diagnosis of AD in the respective daycare centers attended by the patients.

### 2.3. Inclusion and Exclusion Criteria

Inclusion criteria were established for this study:

*Alzheimer’s Disease Group*

This screening met the diagnostic criteria issued by the National Institute on Aging-Alzheimer’s Association workgroups on the diagnostic guidelines for AD. The inclusion criteria for this study included persons aged 60–90 years with clinical symptoms suggestive of AD (clinical diagnosis of AD), with Mental State Examination (MMSE) < 20 points and Montreal Cognitive Assessment (MoCA) < 18 points; Activity of Daily Living (ADL) Scale was impaired (ADL ≥ 22 points); clinical dementia rating (CDR) = 1 point; except for changes in AD, there were no other abnormalities. All the participants were volunteers, free of charge, and signed written consent. The exclusion criteria were the same as those for the normal control group.

*Normal control group*

Normal elderly people in the same period, aged 60–90 years, were selected as the control group (no clinical diagnosis of AD), matched with those in the AD group who were selected and recruited in the outpatient memory department of neurology specialists. Inclusion criteria: no memory deficit or other cognitive impairment and no mental or neurological disease; MMSE ≥ 26 and MoCA ≥ 26. All the participants were volunteers, free of charge, and signed written consent. The exclusion criteria were the same as those for the AD group.

The exclusion criteria included the following: (i) individuals with behavioral alterations or other conditions that prevented sample collection at the time of collection; (ii) participants whose sample collection was hindered by oral alterations incompatible with the technique used; and (iii) thyroid disease, folic acid deficiency, vitamin B12 deficiency, severe anemia and other endocrine and metabolic diseases; and cognitive impairment caused by trauma, infection, drugs or alcoholism, etc.

### 2.4. Procedures

#### 2.4.1. Biological Samples for ApoE Polymorphism Analysis

##### Sample Collection

Saliva samples were collected from participants using the Canvax^®^ Buccal Swab Collection and Stabilization Kit (Canvax Reagents SL, Valladolid, Spain), which includes a swab to scrape the inside of the cheek and collect buccal cells. This process is painless and simple. The swab was then suspended in an Eppendorf tube with stabilization fluid, centrifuged at 13,000 RPM for 5 min, and the supernatant was removed with a pipette, leaving 10–20 μL of residual fluid for preservation until further processing.

##### Processing of Biological Samples

The saliva samples were processed in the laboratory using the Canvax^®^ Buccal Swab Genomic DNA Extraction Kit to purify the DNA (Canvax Reagents SL, Valladolid, Spain). Various buffers (S2, S3, S4 and EB) and proteinase K enzyme were used to ensure efficient DNA purification. Re-suspension solutions were used after the first centrifugation.

##### PCR Amplification

Pantelidis et al. [21] achieved genotypic analysis of the ApoE gene by the polymerase chain reaction (PCR) technique, designing specific primers to amplify ApoE2, ApoE3 and ApoE4 variants at residues 112 and 158. Analyses following their method were performed to identify the ApoE alleles in each individual, using three reactions with different primer combinations to amplify each haplotype. Control primers amplifying regions common to all the haplotypes were used, ensuring specificity with high hybridization temperatures. The PCR products were analyzed by electrophoresis on 2% agarose gels, stained with ethidium bromide and visualized with ultraviolet light. The control primers produced a 785 bp band in all cases, while a 173 bp band indicated an ApoE-specific haplotype. A sample was considered negative for a specific haplotype if the amplification of that haplotype was absent and the 785 bp control band was present.

### 2.5. Statistical Analysis

The statistical analysis carried out included both descriptive and inferential procedures.

Firstly, a description of the data was made, which were tabulated by means of the corresponding frequency distributions, including both the number of individuals (absolute frequencies) and the percentages (relative frequencies).

In the case of qualitative variables, the information was described by taking into account the number and percentage of appearance of the different categories or modalities of the variable. For quantitative variables, the arithmetic mean was used as a summary measure of the information, which was accompanied by the standard deviation or standard deviation as a measure of dispersion that allowed us to assess its representativeness.

As a complement to the descriptive analysis, an inferential study was carried out, with the aim of contrasting the hypotheses relating to the possible existence of differences between individuals clinically diagnosed with pAD and healthy individuals (control group).

Specifically, in the case of quantitative variables, we used the t-test for the difference in the means in independent samples for normal populations. This test requires prior verification of compliance with the normality hypothesis, for which the Kolmogorov–Smirnov and Shapiro–Wilk tests were applied. However, given the size of the sample, and in application of the central limit theorem, it can be assumed that it comes from a normal population.

Finally, in the case of qualitative variables, the chi-square test of independence was used to analyze the relationship between the variables or the z-contrast for the difference in proportions.

The statistical significance of the results was assessed based on the general criterion of a *p*-value of less than 0.05, although cases where significance reached a *p*-value of less than the 1% significance level were also discussed.

All the procedures were carried out with the statistical software IBM SPSS Statistics for Windows, version 26.0 (Armonk, NY, USA: IBM Corp).

## 3. Results

A total of 511 individuals were included in the study population, of whom 260 had a clinical diagnosis of AD, while the remaining 251 were healthy individuals. The 260 subjects diagnosed with pAD were selected from various AFAs, including those located in Soria, Salamanca, León and Ponferrada, as well as from the “Mensajeros de la Paz” residences in Mansilla de las Mulas (León) and La Bañeza (León).

### 3.1. Descriptive Data of the Sample

#### 3.1.1. Socio-Demographic Data

Table 1 presents the socio-demographic characteristics of the study sample, distinguishing between the pAD cases and the control group cases.

The mean age was 76.95 years (±9.72), with those diagnosed with pAD being significantly older (83.94 ± 7.55) than those in the control group (70.28 ± 6.26; *p*-value < 0.0001). Women predominated in both groups, with 76.15% in the cases and 83.67% in the controls (*p*-value = 0.0343). Regarding marital status, 52.30% of cases were widowed, while 63.56% of controls were married (*p*-value < 0.0001).

Regarding the educational level, 74.74% of the cases had a primary education, in contrast to 77.02% of the controls who had a secondary or higher education (*p*-value < 0.0001). The majority resided in urban areas, although the control group represented a significantly higher percentage (95.56%) compared to the cases (72.92%; *p*-value < 0.0001). In addition, 45.60% of cases with pAD reported a family history of the disease, compared to 26.75% in the control group (*p*-value = 0.0002).

#### 3.1.2. Clinical Data

Table 2 presents the clinical characteristics of the study sample, distinguishing between the pAD cases and the control group cases. In our study, significant differences (*p* < 0.05) were found between the pAD group and the control group for the following non-communicable chronic diseases: high blood pressure, hypercholesterolemia, cardiac pathology, and diabetes mellitus (Table 2).

### 3.2. Allele and Genotype Frequencies of ApoE in the Community of Castilla y León (Spain)

This section presents the frequencies of the ε2, ε3 and ε4 alleles, both in the group of cases diagnosed with pAD and in the control group of healthy individuals.

Of the 511 saliva samples analyzed, corresponding to 260 individuals from the case group and 251 individuals from the control group, 8 individuals were recorded without ApoE genotype results due to sample limitations. Consequently, the ApoE frequency analysis was performed on a total of 503 samples, of which 257 were from the pAD case group and 246 were from individuals in the control group. Since the ApoE genotype involves two alleles, twice as many cases in the sample were considered to determine the allele frequency in both groups, as shown in Table 3.

For the first group, the allele frequencies were analyzed for ε3 (0.74) > ε4 (0.22) > ε2 (0.03). These results indicated that, in the group of individuals diagnosed with pAD, the ε3 allele was the most predominant, with a frequency of 74%, followed by the ε4 allele, with a frequency of 22%. The ε2 allele had the lowest frequency in this group, representing 3% of the observed alleles.

The frequencies of the ε2, ε3 and ε4 alleles in the control group of individuals showed slight variations compared to the case group. In this group, the allele frequencies were ε3 (0.86) > ε4 (0.073) > ε2 (0.071). These results indicated that a high frequency of the ε3 allele was observed, constituting 86% of the alleles present. Both the ε2 and ε4 alleles had a frequency of 7%, indicating a relatively even distribution of these alleles in this group.

A chi-2 test of independence was applied from the contingency table, which related the two groups (cases and controls) with the three alleles, resulting in a statistic equal to 49.874, which was associated with a *p*-value of less than 0.0001. Therefore, the null hypothesis that the two variables considered are independent was rejected, and differences were found in the presence of the alleles according to the group of individuals. On the other hand, the frequencies of the genotypes corresponding to the ε2, ε3 and ε4 alleles in the group of individuals diagnosed with pAD and in the group of healthy individuals were presented. These results allowed a detailed view of the distributions of the ApoE genotypes in the population of Castilla y León.

In the case group, a total of 257 saliva samples from individuals diagnosed with pAD were analyzed. The genotypic frequencies of the case group in order from highest to lowest were ε3/ε3 > ε3/ε4 > ε2/ε3 > ε4/ε4 > ε2/ε4, without finding homozygous carriers of the ε2 allele (ε2/ε2). In the control group, a total of 246 samples from healthy individuals without pAD diagnosis were analyzed, with the ApoE genotypic order being ε3/ε3 > ε3/ε4 > ε2/ε3 > ε2/ε4 > ε2/ε2 and without finding, in this case, homozygous carriers of the ε4 allele (ε4/ε4). The majority of the AD and control subjects were ApoE3 carriers. APOE ε3, the most common allele, is believed to have a neutral effect on the disease—neither decreasing nor increasing risk of Alzheimer’s.

Table 4 summarizes the distribution of the genotypes in each of the two groups considered.

In the control group, the majority genotype was homozygous for the ε3 allele, with 72% of cases, followed, to a lesser extent, by the ε3/ε4 and ε2/ε3 genotypes, with respective percentages of 14.2% and 13%.

In the group of cases diagnosed with pAD, the ε3 allele homozygote also stood out, although with a lower percentage of individuals (54.1%), with 34.6% of carriers of the ε3/ε4 genotype.

**Figure 1:**
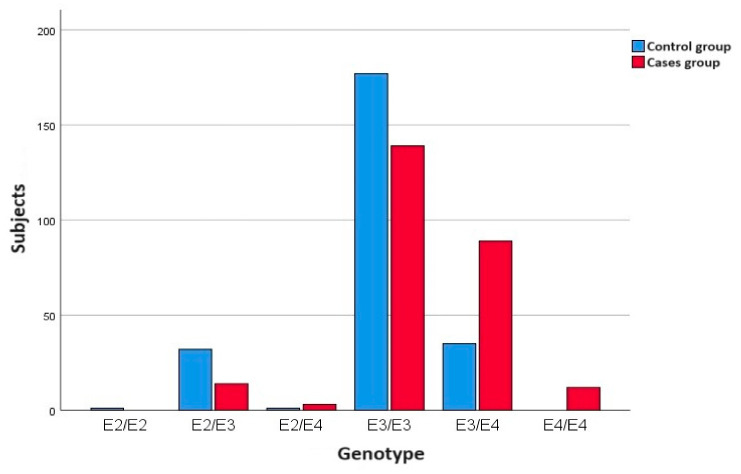
Distribution of genotypes per group.

Figure 1 shows the distribution of the different genotype types in the individuals included in each group.

### 3.3. The ApoE4 Variant in the Population of the Community of Castilla y León

As shown in Table 4, in the group of healthy individuals, there were no homozygous carriers of the ε4 allele, while in the group of cases diagnosed with pAD, there were 12 individuals with this genotype, representing 4.7% of the total. This raised a possible link between the disease and this genotype.

For this purpose, a new contingency table was drawn up relating both groups of individuals, those diagnosed with pAD and the healthy individuals, with the condition of being carriers or not of the ε4/ε4 genotype. Applying a chi-2 test of independence to this table, a *p*-value = 0.0006 was obtained, which leads to the conclusion that the differences in the presence of the ε4/ε4 genotype between the healthy individuals and the subjects with pAD were statistically significant.

Taking into account that the ε4 allele is considered a risk factor for pAD, a classification was made between those individuals with at least one ε4 allele and those individuals without any allele of this type (highlighted in gray in Table 4 versus those without), thus obtaining the results presented in Table 5.

A difference of proportions test was applied to compare the percentage of individuals with an ε4 allele in each group, revealing significant differences between the two percentages (*p*-value < 0.0001). Furthermore, when comparing the risk of suffering pAD in individuals with an allele of this type versus those who did not carry it, through the odds ratio (OR) that relates the complementary probabilities of suffering the disease in both groups, that is, the odds of each group, a value of 3.965 was obtained. This OR indicates that the risk of suffering pAD is almost four times greater in individuals who present at least one ε4 allele than in those who do not have any allele of this type.

## 4. Discussion

The objective of this study was to evaluate the ApoE2 and ApoE4 isoforms as potential biomarkers and to identify individuals at increased genetic risk by polymorphism of the APOE gene. The initial hypothesis was that the presence of the ε2 allele behaves as a protective agent, preventing AD, while the ε4 allele would contrarily have a disruptive role. To achieve this aim, an exhaustive genetic analysis of ApoE variants was carried out in the population of the AFAs of Soria, Salamanca, León and Ponferrada and the residences of “Mensajeros de la Paz” in Mansilla de las Mulas (León) and La Bañeza (León). A total of 511 individuals were evaluated, of whom 260 had been clinically diagnosed with AD and 251 were healthy. The ε4 allele was found to have a higher frequency in the pAD group (22.57%) compared to the control group (7.32%). However, in both cases, the pAD and control groups, the allele with the highest frequency was ε3. APOE ε3, the most common allele, is believed to have a neutral effect on the disease—neither decreasing nor increasing the risk of AD. By means of the chi-2 test, a statistical value of 49.874 was obtained, which was associated with a *p*-value of less than 0.0001 and confirmed a statistically significant allelic difference between the two groups.

Regarding the genotypes, the most frequent one was ε3/ε3 in both groups. However, for the ε4/ε4 genotype, a null frequency was obtained for the control group, while the subjects with pAD presented it at 4.67%. The ε2/ε2 genotype was also not found in any individual with AD but showed a frequency of 0.41% in the healthy participants. The presence of the ε4/ε4 genotype between healthy subjects and pAD patients was likewise found to be statistically significant (*p* equal to 0.0006). Finally, the OR indicated an almost four-fold increased risk of AD in the presence of the ε4 allele compared to those who did not carry it. According to these data, the null hypothesis that ε2 and ε4 did not influence the likelihood of having AD was rejected and the initial hypothesis stating the protective and disruptive role of ε2 and ε4, respectively, was confirmed.

### 4.1. Allele and Genotypic Frequencies at a European Level

In the group of cases with pAD in Castilla y León, there is a notable dominance of the ε3 allele, accounting for 74% of the allele frequencies, followed by the ε4 allele, with 22%. In contrast, the ε2 allele is present in a lower proportion, representing only 3% of the alleles in this set. These frequencies follow similar patterns to those observed in southern European countries such as Greece [21,22], Italy [23] and Portugal [24]. It is particularly interesting to note the strong similarity with the allele frequencies of individuals with AD in southern Greece [22], where the ε3 allele accounts for almost 76%, the ε4 allele 21% and the ε2 allele shows a lower frequency with 3.7%.

In the control group, slight variations in the allele frequencies are observed compared to other European countries. The ε3 allele remains the most prevalent, with a frequency ranging between 60% and 80% in all European countries [22,23,25,26,27]. In Castilla y León, the ε2 and ε4 alleles had a frequency of 7%, showing certain differences compared to other countries where variations in these percentages were observed. The case of the city of Bari, Italy [22], which is characterized by a high frequency of the ε2 allele, reaching 26%, in contrast to the ε4 allele, which shows 9%, is noteworthy. Another study, which showed the allele frequencies in healthy individuals in Italy, specifically in Sardinia and Puglia, highlighted lower percentages of ε2, approximately 5% [28].

In Castilla y León, in both the case and control groups, the most prevalent genotype was ε3/ε3, following the same trend observed in all southern European countries [22,23,24]. Although precise data on the ApoE genotype frequencies in Northern Europe are not available [25,26], an interesting pattern has been observed in Central Europe. The ε2/ε4 genotype stands out with a higher percentage, reaching 57%, compared to the same in Castilla y León, with a frequency of 34% in the case group. This genotype was not found in Portugal [23], neither in the case group nor in the control group.

In the findings, it is notable that in the group of individuals with pAD, the ε2/ε2 genotype was not observed, but the ε4/ε4 genotype was detected with a frequency of 4.6%.

In contrast, in the control group, a frequency of 0.4% was found for ε2/ε2, while no cases of ε4/ε4 were recorded. Both Portugal [24] and Greece [22] had small frequencies of ε4/ε4 in the control group, in contrast to the 0% observed in the results of the present study. Furthermore, in this country, a frequency of 1.4% for the ε2/ε2 genotype was recorded in the group of individuals with AD. When comparing the genotypes in the Bari control group [23] with the results from Castilla y León, subtle differences were also observed. In the city of Bari, a higher percentage of ε2/ε3 was highlighted, reaching 44%, compared to 13% in Castilla y León. This value even exceeded the high ε3/ε3 frequency recorded in all other countries.

### 4.2. Allele and Genotypic Frequencies in Spain

A very limited number of investigations have addressed the analysis of the ApoE allele frequencies in individuals with AD in Spain (Table 6). However, it is important to highlight that the present investigation shows a remarkable consistency in the results concerning the frequencies of the ε3, ε4 and ε2 alleles, in agreement with other existing investigations [24,29].

The study by González et al. [24], who analyzed the ApoE variants in a cohort of individuals with AD in Castilla y León, observed a similar distribution to the results obtained in this work, with a significant frequency of the ε3 allele in both the case group (80.2%) and the control group (79.3%).

Furthermore, the results for the ε4 and ε2 alleles were like the present study, showing a higher allele frequency of ε4 in the case group (19.1%) compared to the control group (12.1%). The percentages of ε2 followed the same trend as in the results obtained, showing a low prevalence in both the case group (0.6%) and the control group (8.6%).

In addition, it is relevant to mention the study by Ibarreta et al. [29], who explored, in individuals diagnosed with AD, the distribution of the ε3, ε4 and ε2 alleles of ApoE in the Community of Madrid (Spain). They observed a significant prevalence of the ε3 allele (60%) in people diagnosed with AD, followed by the frequency of ε4 (34%) and ε2 (6%). These authors, in Madrid, observed frequencies of 67% for ε3 and 27% for ε4 in AD. Regional differences, such as between Madrid and Castilla y León, suggest geographical, ethnic or environmental influences on the ApoE variants.

Detailed exploration of the genetic variants of the ApoE gene in the user population of the Residencias de “Mensajeros de la Paz” and the AFA in Castilla y León reveals a complex network of genetic influences. In this context, it is crucial to consider the various contributions that have shaped the genetic composition of this region. Highlighting the relevance of these contributions, the study by Bycroft et al. [30] on the genomes of 1413 Spanish individuals reveals the genetic dynamics of all the regions of Spain. Castilla y León, due to its strategic geographical location, has received genetic contributions mainly from Western Europe, especially France, and North Africa. In addition, a genetic influence from Italy and small contributions from the Irish population have been highlighted, enriching the genetic diversity of Castilla y León.

### 4.3. ApoE Polymorphism and Alzheimer’s Disease

The results obtained in this study show that the frequency of the ε3/ε4 and ε2/ε4 genotypes was higher in the group of individuals with pAD, with 34.6% and 1.17%, respectively. In contrast, the group of healthy individuals presented lower percentages, with 14.23% and 0.41%, respectively. A higher frequency of the ε4/ε4 genotype is noteworthy in the group of individuals with pAD, reaching 4.67%, while no frequency was recorded in the group of healthy individuals. Nunomura et al. [31] support these findings by indicating that the ApoE4 isoform was significantly higher in the AD group (52.8%) compared to the frequency in the control group (14.5%). Castillo-Reyes et al. [32] also indicated that the ε4 allele exhibited a moderate association with AD, with an OR of 6.5, being more frequent in the cases than in the controls. Consistent with these investigations, the results of the present study reinforce the association between ApoE4 and pAD. An OR of 3.97 was calculated, showing that the risk of pAD is almost four times higher in individuals with at least one ε4 allele compared to those with no ε4 allele.

The strong association of the ε4 allele and the development of AD can be explained by the poor ability of the ApoE4 isoform to clear the Aβ peptide [33,34], which will precipitate and gradually form senile plaques, one of the main events observed in the pathology. The reasons underlying this isoform-specific clearance are still under investigation. A study on the impact of ApoE polymorphism on brain levels [35] observed low amounts of ApoE4 in the brain and CSF at a steady state, probably because its instability causes the molecular machinery to recognize it as a misfolded protein and degrade it. The reduced availability of ApoE4 could negatively influence the degradation efficiency of the peptide, leading to insufficient clearance and gradual aggregation. It has also been reported [36] that the efficiency of ApoE-Aβ complex formation follows the order ApoE2 > ApoE3 >> ApoE4 under physiological conditions and that the ApoE4-Aβ complex is less efficiently cleared at the blood–brain barrier (BBB) [37]. This is because ApoE4-Aβ is redirected from the LDL receptor-related protein 1 (LRP1) to the VLDL receptor (VLDLR), which internalizes the Aβ-ApoE4 complex more slowly than LRP1. The other isoforms clear Aβ by both VLDLR and LRP1 at a much faster rate. Yu-Wen Alvin et al. [38] reported that ApoE4 not only influenced Aβ degradation but also enhanced its synthesis. ApoE4 stimulates a non-canonical MAP kinase signaling pathway that leads to increased transcription of the β-amyloid precursor protein (APP) and a consequent increase in Aβ levels. Other lines of research explain the high frequency of ε4 in AD patients by its tendency to form neurotoxic fragments by proteolytic degradation [39]. In vitro studies have shown that exogenous administration of truncated ApoE4 fragments leads to cell death [40]. Also, in vivo, neurotoxicity, neurodegeneration and behavioral deficits have been observed in transgenic mice [41]. The mechanisms underlying this symptomatology are currently being investigated. Several studies [6,40,42,43,44] have shown that truncated ApoE4 interacts with p-tau and induces intracellular neurofibrillary tangle-like inclusions, disrupts the cytoskeleton, and damages mitochondrial integrity. Other researchers have linked the disruptive role of ApoE4 to its pro-inflammatory properties [45,46,47]. It has been observed that it leads to increased inflammation mediated by astroglia and microglia, generating a persistent inflammatory response, leading to significant brain atrophy and neurodegeneration [48]. Importantly, the association between the ε4 allele and AD is highlighted not only in this study but also in case-control cohort investigations worldwide [31,49,50,51] as well as in the European context [21,22,28] and in the Iberian Peninsula [23]. This supports the evidence of a strong association between the presence of the ApoE4 allele and the prevalence of AD on a global scale.

It should also be noted that the ε2 and ε3 alleles had a higher percentage in the control group. As shown in Table 3, the ε2/ε3 and ε3/ε3 genotypes were more frequent in the control group, registering percentages of 13.01% and 71.95%, respectively. In contrast, in the case group, these genotypes presented lower percentages, being 5.45% and 54.09%, respectively. In addition, the ε2/ε2 genotype was observed in the control group with 0.41%, while it was not detected in the case group. These results are in line with other investigations, such as Ping et al. [51], where the ε2 allele was found to be higher in the control group (0.6%) than in the AD case group (0.5%). Similarly, Goldberg et al. [52] examined the ε2 allele in various neurodegenerative diseases, including AD. They observed that ApoE2 was significantly associated with reduced AD pathology, i.e., it was associated with less PS pathology than ApoE3 and ApoE4, making the ε2 allele protective against the disease, as indicated by the results obtained in the present study and other studies [51,52].

The protective role of ApoE2 may be explained along the same lines as the disruptive role of ApoE4. ApoE2 is the isoform with the highest capacity to clear Aβ and thus prevent plaque formation [33,34]. Deane et al. [37] revealed in their study that the ApoE2-Aβ complex, in contrast to ApoE4-Aβ, is cleared at a high rate via VLDR and LRP1. However, it is not only involved in the metabolism of Aβ but also its catabolism. While ApoE4 stimulates the signaling pathway described in the aforementioned study conducted by Huang et al. [38] (which triggers Aβ production), ApoE2 acts as a protective agent as it has almost no stimulatory capacity of the signaling pathway. This may be because it is a receptor-dependent pathway and it has been observed that the ApoE2 has a markedly lower affinity for its receptor than the remainder isoforms [15]. Several studies [45,46,47] have also highlighted the anti-inflammatory role of ApoE2, which may prevent permanent neuroinflammation associated with the pathology. Meanwhile, Zhao et al. [53] discussed in their study the ability of macrophages to degrade Aβ aggregations through ApoE expression and secretion. A comparison of the efficiency of plaque degradation according to the isoform expressed revealed that macrophages secreting ApoE2 were more efficient than those expressing ApoE4.

Therefore, the APOE risk gene can be considered as a biomarker to be used in the early diagnosis of AD. Thus, the identification of certain APOE polymorphisms could be both preventive and therapeutic, allowing pre-symptomatic intervention and improving the way in which early stages of AD are addressed.

### 4.4. APOE Polymorphism and Potential Therapeutic Approaches

AD is the most common cause of dementia and is projected to increase dramatically in prevalence in the coming decades [5,54]. The pathological picture results in neurodegeneration and a consequent conditioning and impairment of patients’ daily life and social functioning [55]. Therefore, a clinically relevant approach to combat the increasing prevalence of AD is substantial. The modern diagnostic criteria have shifted toward pre-symptomatic recognition by biomarkers [7], combined with clinical and cognitive investigations [56,57].

Since the APOE gene polymorphism has been found to influence cognitive impairment in AD, it is considered a therapeutic target for the pathology. Researchers Serrano-Pozo et al. [58] organize current therapeutic approaches into the following classifications: (i) increasing ApoE levels and its lipidation, (ii) blocking the ApoE–Aβ interaction, (iii) ApoE mimetics, (iv) reducing ApoE levels and (v) gene therapy.

ApoE plays a key role in lipid transport and metabolism by facilitating the formation of high-density lipoproteins responsible for cholesterol and phospholipid transport [59]. These are transferred to ApoE by the lipid transporters ABCA1 and ABCG1, which are regulated by retinoid X receptors (RXR). The low levels of ApoE4 relative to the other isoforms, related to its instability [35], and its low lipidation have led to its upregulation as a potential therapy. Several studies in mice have already [60,61,62] associated ApoE overexpression and lipidation with cognitive improvement and a decrease in Aβ deposits. However, the study by Ghosal et al. [59] with bexarotene (RXR antagonist) in healthy humans had low penetration, did not achieve a large increase in CSF ApoE levels and consequently did not influence Aβ metabolism. Another human study [63] also reported negative results and no improvement in cognitive abilities. Still, both studies do not disqualify bexarotene as a potential drug and justify a phase II/III trial with a larger population and longer periods of time to test its efficacy.

Another potential approach would be to inhibit the ApoE–Aβ interaction. Studies [64,65,66] have shown how treatment with the peptide AB12-28P (homologous to the ApoE and Aβ binding site) reduces amyloid plaques in transgenic mice, tau pathology and behavioral deficits. Liu et al. [64] stress that this approach has a high clinical probability and with effort may hold therapeutic promise.

ApoE mimetics are also being contemplated as a possible therapeutic strategy, which mimic the natural peptide but promise advantages such as greater structural strength or proteolytic stability [67,68]. Krishnamurthy et al. [69] conducted a murine study with transient treatment with CN-105 from 14–18 weeks of age and reported a reduction in Aβ plaque and an improvement in memory deficits. The same procedure at 25–28 weeks of age did not have such noticeable effects. The efficacy and feasibility of CN-105 were evaluated in a phase II clinical study in older adults [70].

Anti-APOE immunotherapy with monoclonal antibodies is also promising [58]. Fan Liao et al. [71] administered anti-APOE HJ6.3 to mice and reported that it prevented amyloid deposition, deposit formation and occasional plaque clearance. Another study applying the same antibody intraperitoneally to mice [72] revealed a dramatic decrease in amyloid deposition and changes in the microglial responses around plaques.

Downregulation of ApoE4 by antisense nucleotides [73] is also being studied as a possible therapy. However, a study in transgenic mice [74] showed that ApoE reduction before plaque formation markedly affects the onset of Aβ pathology, while ApoE reduction after plaque formation only modulates the plaque size and toxicity. Interpreting these results, Serrano-Pozo et al. [58] suggest a possible preventive rather than therapeutic use of ApoE silencing.

Finally, the possibility of gene therapies as a therapeutic strategy should be mentioned. An in vitro [75] study performed the conversion of ApoE4 into ApoE3 by gene editing, with promising results: fewer neurotoxic fragments and decreased AB production, tau phosphorylation and degeneration of gabanergic neurons. Hudry et al. [34] in their study chose to use gene transfer with an adeno-associated viral vector in transgenic mice. They found that the administration of ApoE2 by gene transfer prevented plaque formation, increased Aβ output to plasma and attenuated synapse loss. Both approaches appear promising, and the field of gene therapy needs phase II/III clinical trials but could be another potential strategy for plaque removal and synaptic restoration [34].

## 5. Conclusions

In the present study, a higher presence of the ε4 allele was found in the pAD group than in the control group. It was also observed that the ε2/ε2 genotype was not found in any individual with pAD but was found in the healthy subjects and that the opposite was observed for the ε4/ε4 genotype. The OR indicated a risk four times greater of having AD if having the ε4 allele. The significant association between the ε4 allele and the development of AD was confirmed in this experiment as well as at the national and European level. In contrast, the ε2 allele has been identified as a protective factor, having a higher percentage in the control group, by this and other studies.

The molecular mechanisms that confer a disruptive and protective role to ApoE4 and ApoE2, respectively, are still being studied. The formation of senile plaques and isoform-dependent differential Aβ deposition are considered as a key factor in neurodegeneration. In addition, ApoE mechanisms independent of dysfunctional Aβ clearance are investigated, such as susceptibility to proteolysis and the implications of the resulting neurotoxic fragments or neuronal changes long before the actual formation of neurofibrillary tangles and neuropil threads [76]. Other approaches explore the role of isoform-dependent neuroinflammation and its implications for the pathological picture of the disease. However, these are only a few of the many lines of research currently being pursued and much work remains to be performed to understand the molecular mechanisms of the ApoE protein. The demonstrated relation between the different isoforms and the likelihood of developing AD has led to its consideration as a biomarker and potential pre-symptomatic therapy. There are many possible approaches to ApoE as a therapy that have shown promising results in mice, yet translation into clinical trials is complicated and remains a challenge for the future.

## Figures and Tables

**Table 1:** Socio-demographic information of the study participants per group.

Characteristics		pAD Case Group	Control Group	*p*-Value
Age mean (SD)		76.95 (9.72)	70.28 (6.26)	<0.0001
Gender *n* (%)	Men	62 (23.85)	41 (16.33)	0.0343 *
	Women	198 (76.15)	210 (83.67)	
Civil status *n* (%)	Single	12 (5.02)	22 (8.91)	<0.001 ***
	Married	100 (41.84)	157 (63.56)	
	Widowed	125 (52.30)	50 (20.24)	
	Divorced	1 (0.42)	13 (5.26)	
	Separated	1 (0.42)	5 (2.02)	
	Not available	21 (-)	4 (-)	
Educational level *n* (%)	Without studies	7 (3.68)	0 (0)	<0.001 ***
	Primary	142 (74.74)	54 (22.98)	
	Secondary	16 (8.42)	98 (41.70)	
	Higher	25 (13.16)	83 (35.32)	
	Not available	70 (-)	16 (-)	
Residential area *n* (%)	Rural	65 (27.58)	11 (4.44)	<0.001 ***
	Urban	175 (72.92)	237 (95.56)	
	Not available	20 (-)	3 (-)	
Family history of AD *n* (%)	Yes	88 (45.60)	65 (26.75)	0.002 **
	No	101 (52.30)	174 (71.60)	
	Unknown	4 (2.10)	4 (1.65)	
	Not available	8 (-)	8 (-)	

Abbreviations: pAD: probable Alzheimer’s disease. SD: standard deviation. (-) no data. Values are expressed as the mean (SD) for quantitative variables and as the frequency (percentage) for categorical variables. z-test for gender and residential area; chi-2 test for variables of civil status, educational level, and family history of AD. Differences were statistically significant at *p* < 0.05. * *p* < 0.05; ** *p* < 0.01; *** *p* < 0.001.

**Table 2:** Clinical information of the study participants per group.

Chronic Diseases		pAD Group (n = 240)		Control Group (n = 248)		*p*-Value
		Frequency	%	Frequency	%	
High blood pressure (≥140/90 mmHg)	Yes	94	39.17	61	24.60	0.0005 ***
	No	146	60.83	187	75.40	
Hypercholesterolemia (LDL-cholesterol > 190 mg/dL)	Yes	73	30.42	91	36.69	0.1422
	No	167	69.58	157	63.31	
Cardiac pathology European Society of Cardiology (ESC) criteria	Yes	49	20.42	22	8.87	0.0003 ***
	No	191	79.58	226	91.13	
Diabetes mellitus blood glucose ≥ 200 mg/dL	Yes	47	19.58	18	7.26	<0.0001 ***
	No	193	80.42	230	92.74	

Abbreviation: pAD: probable Alzheimer’s disease; enumeration data were subjected to the Chi-square test and described as percentages. Differences were statistically significant at *p* < 0.05. *** *p* < 0.001.

**Table 3:** Allele frequency and percentage per group.

Allele	pAD Case Group		Control Group	
	Frequency	%	Frequency	%
ε2	17	3.31	35	7.11
ε3	381	74.12	421	85.57
ε4	116	22.57	36	7.32
Total	514	100	492	100

Abbreviation: pAD: probable Alzheimer’s disease.

**Table 4:** ApoE genotype profile per group.

Genotype	pAD Case Group		Control Group	
	Frequency	%	Frequency	%
ε2/ε2	0	0	1	0.41
ε2/ε3	14	5.45	32	13.01
ε2/ε4	3	1.17	1	0.41
ε3/ε3	139	54.09	177	71.95
ε3/ε4	89	34.63	35	14.23
ε4/ε4	12	4.67	0	0
Total	257	100	246	100

Abbreviation: pAD: probable Alzheimer’s disease.

**Table 5:** Distribution of the ε4 allele by group.

ALELO	pAD Case Group		Control Group		Probability		Odds
	Frequency	%	Frequency	%	pAD	Control	
ε4^+^	208	40.47	72	14.63	0.743	0.257	2.889
ε4^−^	306	59.53	420	85.36	0.421	0.578	0.729
Total	514	100	492	100	OR		3.965

Abbreviation: pAD: probable Alzheimer’s disease; OR: odds ratio ε4^+^: with at least one Apoε4 allele; ε4^−^: without any Apoε4 allele.

**Table 6:** ApoE allele and genotype frequencies of individuals diagnosed with Alzheimer’s disease and healthy subjects in Spain.

Population	Group	n	Alleles			Genotypes						
			E2	E3	E4	E2/E2	E2/E3	E2/E4	E3/E3	E3/E4	E4/E4	Reference
**Central region**	**AD**	71	6	67	27	-	-	-	-	-	-	[28]
	**Control**	50	5	92	4	-	-	-	-	-	-	
**South region**	**AD**	81	0.6	80.2	19.1	0	1.2	0	64.2	30.9	3.7	[23]
	**Control**	29	8.6	79.3	12.1	0	17.2	0	65.5	10.3	6.9	
**Northwest region**	**AD**	251	3	74	22	0	5.4	1.2	54.1	34.6	4.6	Our study
	**Control**	250	7.1	86	7.3	0.4	13	0.4	71.9	14.2	0	
**Spain (total)**	**AD**	403	9.6	221.2	68.1	0	6.6	1.2	118.3	65.5	8.3	
	**Control**	329	20.7	257.3	23.4	0.4	30.2	0.4	137.4	24.5	6.9	

Abbreviation: AD: Alzheimer’s disease; n: sample.

## Data Availability

The original contributions presented in the study are included in the article; further inquiries can be directed to the corresponding author.

## Appendix A

Appendix A is the Strengthening the Reporting of Observational Studies in Epidemiology (STROBE) statement.
